# Decoration of ZnO Nanorods with Coral Reefs like NiO Nanostructures by the Hydrothermal Growth Method and Their Luminescence Study

**DOI:** 10.3390/ma7010430

**Published:** 2014-01-15

**Authors:** Mazhar Ali Abbasi, Zafar Hussain Ibupoto, Mushtaque Hussain, Galia Pozina, Jun Lu, Lars Hultman, Omer Nur, Magnus Willander

**Affiliations:** 1 Physical Electronics and Nanotechnology Division, Department of Science and Technology (ITN) Campus Norrköping, Linköping University, Norrköping SE-60174, Sweden; E-Mails: zafar.hussain.ibupoto@liu.se (Z.H.I.); mushatque.hussain@liu.se (M.H.); omer.nour@liu.se (O.N.); magnus.willander@liu.se (M.W.); 2 Thin Film Physics Division, Department of Physics, Chemistry and Biology (IFM), Linköping University, Linköping SE-58183, Sweden; E-Mails: galia@ifm.liu.se (G.P.); junlu@ifm.liu.se (J.L.); larhu@ifm.liu.se (L.H.)

**Keywords:** ZnO nanorods, NiO nanostructure, composite nanostructures, defect states, cathodoluminescent

## Abstract

Composite nanostructures of coral reefs like p-type NiO/n-type ZnO were synthesized on fluorine-doped tin oxide glass substrates by hydrothermal growth. Structural characterization was performed by field emission scanning electron microscopy, high-resolution transmission electron microscopy, and X-ray diffraction techniques. This investigation shows that the adopted synthesis leads to high crystalline quality nanostructures. The morphological study shows that the coral reefs like nanostructures are densely packed on the ZnO nanorods. Cathodoluminescence (CL) spectra for the synthesized composite nanostructures are dominated mainly by a broad interstitial defect related luminescence centered at ~630 nm. Spatially resolved CL images reveal that the luminescence of the decorated ZnO nanostructures is enhanced by the presence of the NiO.

## 1. Introduction

ZnO has been widely used for developing optoelectronics devices due to its versatile physical characteristics including wide direct bandgap of 3.37 eV, high exciton binding energy of 60 meV, and ability to transmit light [1]. ZnO nanomaterial is potentially used in different applications such as optoelectronics, sensors, actuators, optics, piezoelectric nanogenerator, and biomedical sciences [2,3]. Due to favorable properties exhibited by ZnO nanostructures, new trends can be followed in the application of heterojunction based nanomaterials with combined usability for the specific purpose such as UV-blue light emitting diodes (LEDs) [4] and UV photo detectors [5]. Thus, ZnO is a promising alternative material to GaN, which has been extensively utilized in the LEDs operating in the UV-blue spectral window for more than two decades [4,6,7]. Despite several advantages of GaN, it is difficult to grow GaN nanowires for the development of nano-LEDs. Recently, a number of p-n heterojunctions have been fabricated using n-type ZnO nanowires with p-type GaN or silicon [8–10].

Several growth techniques have been used for the fabrication of ZnO nanostructures [6–11]. Among them, the hydrothermal growth method is considered as cheap, simple, achievable at low temperature and environmentally friendly [11,12]. This method yields a material, which contains point defects contributing to defect-assisted emission under normal conditions [12]. Controlled morphology and highly oriented ZnO nanorods (NRs) can be achieved by using a seed layer of ZnO nanoparticles [10]. Since the fabrication of high-quality p-type ZnO is still a challenging task, an alternative n-ZnO/p-based heterojunction has been explored [13]. Besides other p-type semiconducting materials, NiO attracts much interest because of some of its attractive properties such as wide direct bandgap of ~3.6–4.0 eV at room temperature, high hole mobility and low lattice mismatch with ZnO. Also, the synthesis of NiO is simple either in the form of thin films or nanostructures depending on the growth techniques. NiO nanostructures can be synthesized by the electrochemical method [14], sol gel technique [15], spray deposition method [16] and thermal evaporation [17]. The synthesis of NiO nanostructures can also be done by the simple, cheap, and low temperature aqueous chemical growth techniques [18,19]. By exploiting the properties of NiO nanostructures in combination with ZnO nanostructures quite a few p-n heterojunctions have been reported [20]. Few studies on the fabrication of p-type NiO/n-type ZnO heterojunctions with different techniques in which the interface properties have been investigated [21]. NiO/ZnO composites have been studied extensively as p-n diode, UV photo detectors [22,23] and LEDs [24,25]. In addition to this, the luminescence study of ZnO has been widely explored by coating different materials on ZnO nanostructures such as MgO [26,27] Al_2_O_3_ [28] and SnO_2_ [29,30], along with metals such as Au [31], Ag [32], and Pt [33] in order to enhance the near band edge (NBE) emission. However, fewer reports are available on the enhancement of the visible emission of ZnO by coating materials [34]. We believe that the composite nanostructures of NiO/ZnO can pave the way for the fabrication of white LEDs due to the increased visible broad emission related to the defect states in the ZnO and defects at the interface of the heterojunction. The grown NiO/ZnO composite nanostructures have revealed an enhanced red-shift in the visible emission compared to pure NiO and pure ZnO nanostructures, which might have resulted from a successful localized alloying of NiO on the ZnO nanorods that enhanced oxygen related interstitial defects.

Since ZnO nanostructures are of potential to many optoelectronic devices like LEDs, UV detectors *etc.* and unintentionally grown NiO is p-type, therefore it will be of interest to investigate the luminescence properties of ZnO nanorods decorated with NiO nanostructures. Thus, the presented composite nanostructures can be beneficial for further development of optoelectronic devices due to improved intensity of visible luminescence.

## 2. Results and Discussion

**Figure 1.**
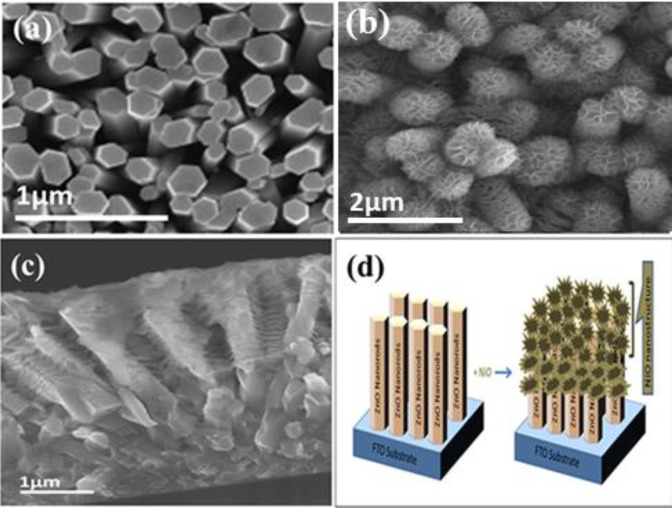
(**a**) SEM image of pure ZnO nanorods; (**b**) SEM image of ZnO nanorods decorated with NiO nanostructures; (**c**) Cross sectional SEM image of the ZnO nanorods covered with NiO nanostructures; (**d**) Schematic diagram of composite NiO/ZnO heterojunction formation.

Figure 1 shows SEM images of the p-NiO/n-ZnO composite nanostructures fabricated on FTO glass substrate together with a schematic diagram of the structure. As shown in Figure 1a, the ZnO NRs are well aligned and vertically oriented to the surface of the substrate and having a diameter of around 200–300 nm. Figure 1b demonstrates that the ZnO NRs are uniformly covered with NiO forming flower-like arranged coral reefs with an average thickness of ~50–80 nm. The possible reaction mechanism for the preparation of the ZnO nanorods using zinc nitrate hexahydrate and hexamethylenetetramine as primary precursors is described below [35]:

$$C_{6}H_{12}N_{4}+6H_{2}O\to 4{\text{NH}}_{3}+6\text{HCHO}$$ (1)

$${\text{NH}}_{3}+H_{2}O\to {\text{NH}}_{4}{}^{+}+{\text{OH}}^{-1}$$ (2)

$${\text{Zn}}^{2+}+2{\text{OH}}^{-1}\to \text{Zn}{\left(\text{OH}\right)}_{2}$$ (3)

$${\text{Zn(OH)}}_{2}\to \text{ZnO}+H_{2}O$$ (4)

However, the decoration of ZnO nanorods with the NiO nanostructures was achieved by using nickel acetate and hexamethylenetetramine precursors in identical fashion as described above for ZnO nanorods:

$$C_{6}H_{12}N_{4}+6H_{2}O\to 4{\text{NH}}_{3}+6\text{HCHO}$$ (5)

$${\text{NH}}_{3}+{\text{ H}}_{2}O\to {\text{NH}}_{4}{}^{+}+{\text{OH}}^{-1}$$ (6)

$${\text{Ni}}^{2+}+2{\text{OH}}^{-1}\to {\text{Ni}(\text{OH})}_{2}$$ (7)

Here, the nickel hydroxide nanostructures are accumulated on the surface of the ZnO nanorods. Further annealing of nanorods with nickel hydroxide nanostructures results in the formation of NiO nano-flakes due to the dehydration of nickel hydroxide as described below:

$${\text{Ni}(\text{OH})}_{2}\to \text{NiO}+H_{2}O$$ (8)

**Figure 2.**
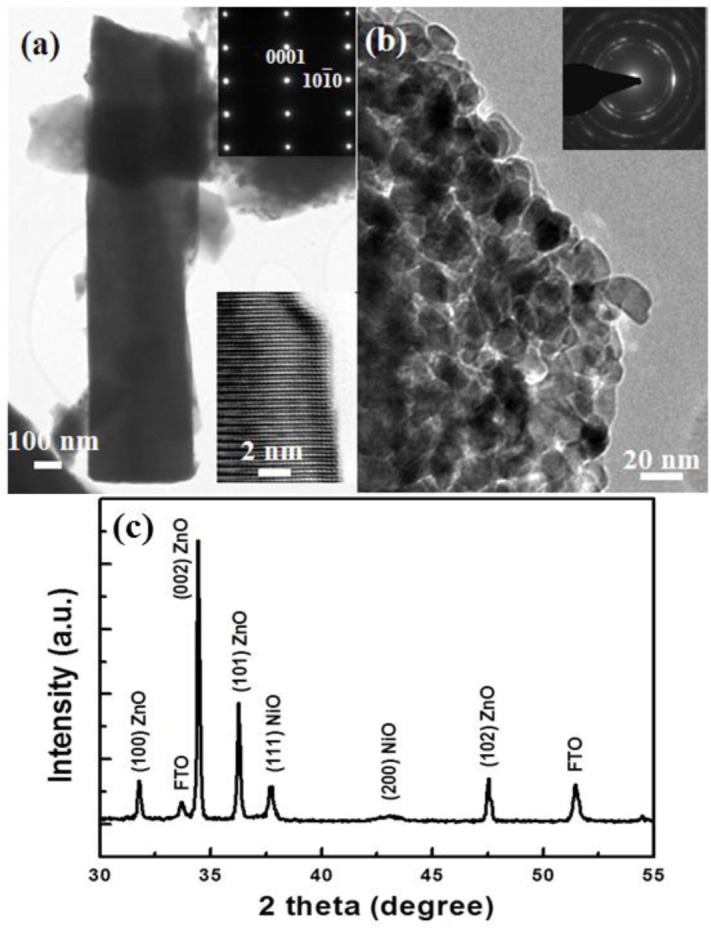
(**a,b**) TEM images with corresponding selected area electron diffraction (SAED) patterns of ZnO NR and NiO, respectively; (**c**) X-ray diffraction pattern for composite nanostructures of NiO/ZnO.

Figure 1c demonstrates a cross sectional SEM image of the ZnO nanorods covered with NiO nanostructures. Figure 1d illustrates the schematic diagram for the complete growth process of ZnO nanorods and decoration with NiO nanostructures on a FTO glass substrate. Figure 2a shows a high resolution transmission electron microscope (HRTEM) image together with the corresponding selected area electron diffraction (SAED) pattern of a single ZnO nanorod. It can be seen that the ZnO NR exhibits single-crystal characteristics with a wurtzite crystal structure and is directed along the [001] direction. The HRTEM image of the ZnO NR is shown in the inset of Figure 2a and the SAED analysis gave the lattice spacing as 0.26 nm for ZnO NRs which corresponds to the (002)-lattice spacing of the hexagonal structure of single crystal ZnO. The obtained HRTEM results thus confirm that a high crystalline quality of ZnO NRs has been achieved. Further, a HRTEM study was also carried out for the NiO nanostructures on the ZnO nanorods and the obtained results demonstrated that the NiO nanostructures consist of face centered cubic nanoparticles with a diameter of ~20 nm as shown in Figure 2b.

The XRD 2θ-ω spectrum of the NiO/ZnO composite nanostructures is shown in Figure 2c. Three of the observed diffraction peaks indicated by stars are indexed to reflections of the (111) and (200) planes of face centered cubic crystal structure of NiO (JCPDS No. 47-1049). Moreover, diffraction peaks of ZnO NRs were also observed and these peaks can be assigned to the hexagonal wurtzite crystal phase of ZnO (JCPDS card No. 36-1451). As it can be seen from the XRD spectrum, a (002) peak is very intense, which demonstrates that the preferred growth direction of ZnO nanorods is along the c-axis. Other peaks are reflections from the FTO substrate. The combined results of the XRD and the HRTEM analysis show a rather high crystalline quality of both the ZnO and the NiO nanostructures.

**Figure 3.**
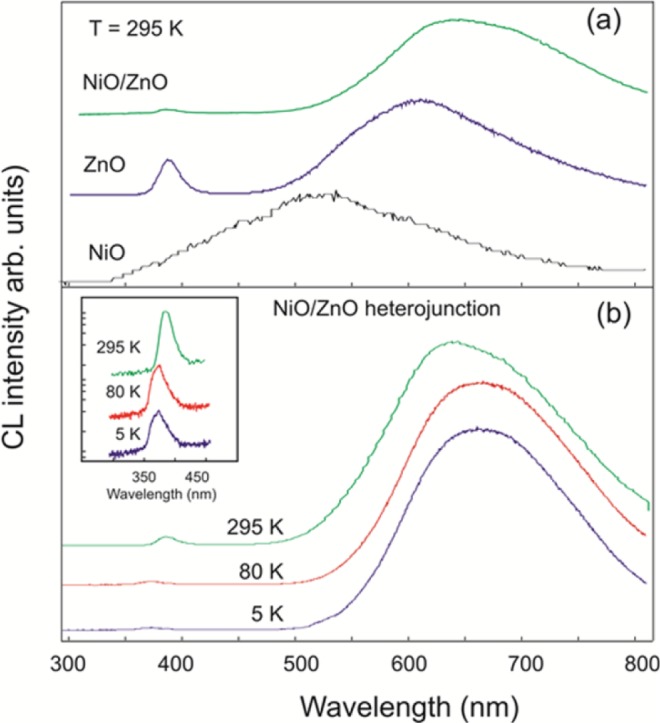
(**a**) Normalized CL spectra at room temperature for NiO, ZnO and composite nanostructures; (**b**) Normalized CL spectra for NiO/ZnO heterostructures shown at three different temperatures. Inset shows the effect of temperature on near band gap luminescence.

Figure 3a shows a comparison of the room-temperature cathodoluminescence (CL) spectra of pure NiO, pure ZnO and the NiO/ZnO nanostructures grown separately on identical FTO glass substrates giving no contribution to the CL spectrum. The spectra are normalized and vertically shifted for the clarity. The pure NiO nanostructures showed a very weak broad emission with a maximum in the green region, which is in good agreement with reported work [36,37]. It is worth mentioning that the CL intensity acquired at the same conditions was weaker by a few orders of magnitude for NiO than for ZnO or the composite nanostructures. The CL spectra of ZnO NRs are dominated by two typical emissions centered at 383 nm and 610 nm correlated to the near band gap (NBG) and to point defects transitions, respectively [38,39]. Relative intensities for these two bands were similar for all the investigated ZnO NRs. In comparison to the pure ZnO NRs sample, the CL spectra of the NiO/ZnO composite nanostructure demonstrate a significant enhancement of the defect emission at 650 nm compared to the ZnO NBG line at 383 nm (see Figure 3). Also, the composite nanostructures showed enhanced red shift of the peak position for the visible emission compared to pure NiO and ZnO nanostructures. It has been proven that the orange/red emission in ZnO is due to the oxygen interstitial which appears in the range of 620–690 nm, however in the present case of NiO/ZnO composite nanostructures, a small shift in the visible emission centered at 650 nm is observed due to enhanced oxygen related interstitial defects [40,41]. Normalized temperature dependent CL spectra of the composite NiO/ZnO nanostructure are shown in Figure 3b. With increasing temperature from 5 to 295 K, the NBG luminescence showed a typical red shift from 370 nm to 385 nm as illustrated in the inset of Figure 3b. We have also observed that the relative intensity between the visible and the NBG luminescence changes with temperature. The enhancement of the orange-red emission at ~650 nm compared to the NBG line is clearly seen at 5 K. This low energy luminescence tail can be explained by recombination between centers involving deep and shallow (localized states) level defects. With increasing temperature, shallow defects will be thermalized and thus do not contribute to the CL spectrum, which results in blue shift of the CL peak position [41,42].

**Figure 4.**
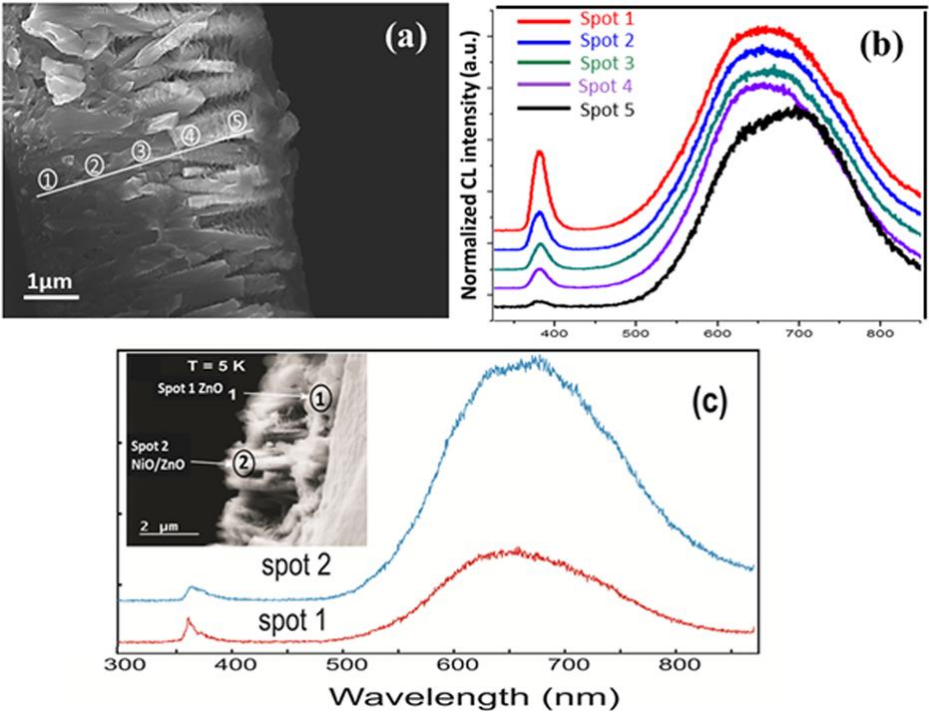
(**a**) Cross-sectional SEM image for CL of NiO/ZnO composite nanostructures; (**b**) Normalized CL spectra for NiO/ZnO heterostructures at five different spots; (**c**) CL spectra for ZnO and NiO/ZnO heterojunction at 5K. Inset showing the cross sectional SEM view for these CL spectra.

In addition to this, more investigations have been done for the cross sectional view of the composite nanostructures at 5 K and at 295 K as shown in Figure 4. An electron beam was located at five different spots starting from bare ZnO NRs located down close to the substrate indicated as spot 1 and moving gradually upwards the ZnO NR part covered with coral reefs like NiO nanostructures indicated as spot 2 to spot 5, respectively. For each spot, we kept the acquisition parameters unchanged and only the electron beam position was moved. Figure 4b illustrates the normalized CL spectra of these five spots. The relative intensity of the visible luminescence for the nanocomposite is much higher than the NBG emission as seen in Figure 4b. Figure 4c represents the comparative CL spectra of ZnO NRs and NiO/ZnO nanostructures at 5 K denoted by spot 1 and spot 2, respectively. Since the experimental conditions were the same under measurements at these two points, one could note that the relative CL intensity is higher for the NiO/ZnO heterojunction (*i.e.*, for the CL spectrum taken at point 2 in Figure 4c). This CL behavior was confirmed for different places over the samples and also at low (5 K) and room temperature. Table 1 represents the quantitative ratio between the relative CL intensities at room temperature. Spot 1 is placed on the bare ZnO nanorods and then spot 2 to spot 5 are in the direction from ZnO to NiO/ZnO heterojunction, such that the amount of NiO nanostructures is increasing gradually. At spot 1, the quantitative ratio is only 2.5 but with a change in the beam position from spot 1 to spot 2 along a straight line without changing any other parameters, we observed a significant change in the quantitative ratio which is found to be double the quantitative ratio at spot 1. The ratio for spot 5 is 18, which is almost eight times higher than that for spot 1, *i.e.*, bare ZnO. Since NiO contributes only negligibly to the CL intensity (as seen in Figure 3), we can conclude that enhanced defect related luminescence from the composite nanostructure is due to the influence of the p-type NiO on n-type ZnO and can be related to hole injection and simultaneous recombination of electrons and holes [43].

## 3. Experimental Section

Commercially available fluorine doped tin oxide (FTO) coated glass substrate was used for the synthesis of ZnO NRs by the hydrothermal growth method through two steps. In the first step, a seed solution of ZnO nanoparticles was spin coated on the substrate and subsequently annealed at 120 °C. The seed solution of ZnO nanoparticles was prepared by dissolving 274 mg of zinc acetate dihydrate in 125 mL of methanol and solution was left for constant stirring of 2 h. At the same time 109 mg of potassium hydroxide were dissolved in 65 mL of methanol separately and mixed with the zinc acetate dihydrate solution drop wise at the temperature of 60 °C [10]. In the second step, the annealed substrates were dipped vertically in the glass beaker containing equimolar (0.075 M) solution of zinc nitrate hexahydrate and hexamethylenetetramine. After that the beaker was kept in preheated electric oven at 90 °C for duration of between 4 and 6 h. Then, NiO nanostructures were fabricated on the ZnO nanorods also using the two-step hydrothermal growth method. In the third step, a seed solution of nickel acetate was spin coated on the ZnO nanorods and then nanorods were dipped in equimolar solution of nickel acetate and hexamethylenetetramine at 90 °C in the preheated electric oven for 4 h. Finally, the sample of NiO nanostructures grown on the ZnO NRs was annealed at 450 °C for duration of 3–4 h to get the complete conversion of nickel hydroxide into NiO crystalline phase. Both pure NiO and ZnO samples were also grown on FTO coated glass for comparison. The morphology and the structural properties of the nanofabricated heterostructures were studied by field emission scanning electron microscopy (FESEM) equipped with energy-dispersed X-ray spectroscopy (EDS) detector and by high-resolution transmission electron microscopy (HRTEM), respectively. HRTEM was carried out using an FEI Tecnai G2 TF20 UT (FEI, Eindhoven, The Netherlands) with a field emission gun working at 200 kV and a point resolution of 1.9 Å. Crystal qualities of NiO/ZnO nanorods was studied by X-ray diffraction (XRD, Oxford, United Kingdom) technique using a standard diffractometer. Cathodoluminescence (CL) technique was used for the investigation of luminescence properties of the proposed heterostructures based on p-type NiO/ZnO nanostructures. CL spectra were measured using a Mono CL 4 system integrated with a LEO 1550 Gemini SEM (Zeiss, Oberkochen, Germany) and equipped with a fast CCD detection system and with a Peltier cooled photomultiplier tube for signal acquisition at an accelerating voltage of 10 kV. For low temperature CL measurement, a liquid-He-cooled stage has been used.

## 4. Conclusions

Closely-spaced and well-aligned coral reef-like composite nanostructures of p-type NiO on n-type ZnO NRs were fabricated on FTO glass substrate by the relatively cheap and simple hydrothermal growth method. CL study has shown an enhancement of the luminescence in the visible region and the effect is more pronounced at low temperatures. Moreover, the quantitative analysis of the cross-sectional study confirmed the enhancement in the visible emission of grown composite nanostructures as much as eight times more than pure ZnO. The quantitative analysis and the obtained results indicate that the p-type NiO/n-type ZnO composite nanostructure is a promising material for the development of optoelectronic nanodevices.

## Figures and Tables

**Table 1.** Quantitative ratio of relative cathodoluminescence (CL) intensities at room temperature.

Spot	Quantitative ratio
1	2.5
2	5
3	6.8
4	9.6
5	18
